# In Memoriam—Dr. Francis Peabody

**Published:** 1897-03

**Authors:** 


### OBITUARY.

### In Memoriam.

DIED, Saturday, January 30, 1897, at 3 A. M., at Ft. Myer, Florida, Dr. Francis Peabody.

Dr. B. O. Doyle sends the following biographical sketch of the late Dr. Francis Peabody, accompanied by the resolutions of respect, sympathy and condolence passed at a special meeting of the dentists of Louisville, Ky., and adjacent cities:

"Dr. Peabody was born in Boston, Mass., January 22, 1833. He came to Louisville, Ky., when a young man, and began the study of dentistry with his uncle, Dr. W. H. Goddard, who was one of Louisville's distinguished dentists in the early days. After studying with his uncle for some time, he went to Cincinnati and graduated in his profession from the Ohio Dental College.

"During the war he lived near Nashville, and while he took no active part on either side, he sympathized with the 'lost cause.' During the war his first wife died; he has a grown daughter by this union, now residing in Boston.

"At the close of the war Dr. Peabody returned to Louisville and again began the practice of his profession with his uncle, Dr. Goddard. He remained here until 1871, when he went to Brazil, and practiced his profession in Rio Janeiro. He again returned to Louisville, and remained here until his death. In 1882 he married Miss Nannie E. Williams, who, with two interesting children, survive him.

"Dr. Peabody was, perhaps, one of the best-known dentists in Kentucky. He was elected to the Faculty of the Louisville College of Dentistry in 1888, and has been President of that institution for about six years. He has been President of the Kentucky State Dental Association, and Chairman of the Board of Censors, both very distinguished honors. He was a member of the National Association of Dental Faculties, American Dental Association, Southern Dental Association, and Mississippi Valley Dental Association. He was elected President of the Falls City Dental Club last year. He was an enthusiastic student and able teacher, and a practitioner of rare skill and ability."

---

"LOUISVILLE, KY., February 1, 1897.

"At a special meeting of the Falls Cities Dental Club, held at Dr. Dunn's office, February 1, 1897, called for the purpose of taking suitable action upon the death of Dr. Francis Peabody, a committee was appointed to draft resolutions which were submitted as follows:

"WHEREAS, It has pleased an All-wise Providence to take from among us our beloved friend and President, Dr. FRANCIS PEABODY, be it, therefore,

"*Resolved*, That we, the members of the Falls Cities Dental Club, recognize and deeply feel the great loss which the profession of Dentistry, and our Club in particular, has sustained in Dr. Peabody's death; and be it further

"*Resolved*, That he was a true and loyal friend, generous, kind and just in his broad, filial nature; ever ready to give encouragement to his younger associates and to sacrifice himself for the weal of others. Zealous in the work of advancing and maintaining the elevation of his profession, and protecting it with dignity from invasion by anything which threatened to degrade it, and further be it

"*Resolved*, That we offer our sympathy and condolence to his family, with the assurance that their bereavement is ours also.

"*Resolved*, That we attend the funeral in a body, and further,

"*Resolved*, That these resolutions be spread upon a memorial page of our Record; that a copy be sent to his family, and that they be published in at least one dental journal.

H. B. TILESTON,

B. OSCAR DOYLE,

ED. M. KETTIG, *Committee.*"

CHAS. E. DUNN,

J. H. BALDWIN.

